# Predictive value of exosomes and their cargo in drug response/resistance of breast cancer patients

**DOI:** 10.20517/cdr.2019.90

**Published:** 2020-03-19

**Authors:** Heidi Schwarzenbach, Peter B. Gahan

**Affiliations:** ^1^Department of Tumor Biology, University Medical Center Hamburg-Eppendorf, Hamburg 20246, Germany.; ^2^Fondazione “Enrico Puccinelli” Onlus, Perugia 06123, Italy.

**Keywords:** Breast cancer, exosomes, exosomal DNA, mRNA, non-coding RNAs, microRNAs, lncRNAs

## Abstract

Exosomes are small extracellular vesicles engaged in intercellular communication in both healthy and tumor cells. When released by the primary tumor, they transfer their cargo including nucleic acids, proteins, and lipids to target cells, thus modulating the character and fate of the recipient cells. By propagating their oncogenic content, exosomes are able to promote tumor progression, angiogenesis, metastases, and drug resistance. Their functions as delivery vehicles of biological material make exosomes promising biomarkers for the early prediction of disease progression and drug resistance in breast cancer, as well as for therapeutic targeting of molecules to treat this deadly disease. In the present review, we accentuate the relevance of exosomes as vehicles of prognostic and predictive markers and target molecules, and describe their potential therapeutic applications as drug cargo suppliers. We made an extensive literature research to clarify the association of their cargo, including exosomal DNA and RNA molecules, with the propagation of drug resistance.

## Introduction

**Figure 1:**
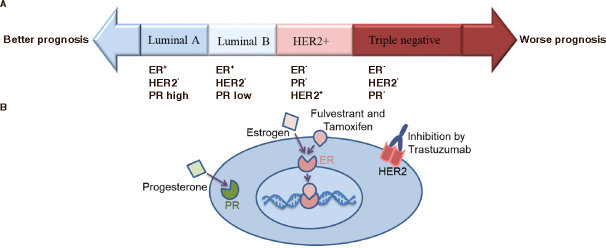
A: Breast cancer comprises four subtypes defined by the positive (+) and negative (-) status of the ER, PR, and HER2; B: The breast cancer cell is positive for all three receptors. Cell proliferation is controlled by the ER and HER2 and is inhibited by the competitive binding of either fulvestrant or tamoxifen to the ER or trastzumab to HER2. ER: estrogen receptor; PR: progesterone receptor; HER2: human epidermal growth factor receptor 2

Breast cancer is comprised of four subtypes that can be defined by either the positive or the negative status of the estrogen receptor (ER), progesterone receptor (PR), and human epidermal growth factor receptor 2 (HER2). Triple-negative and HER2-positive tumors are associated with a poor prognosis, a more aggressive clinical outcome, and a higher risk of relapse than luminal-like tumors that are positive for the hormone receptors. When the breast cancer cell is positive for all three receptors, cell proliferation is controlled by the ER and HER2 and, in consequence, can be inhibited by the competitive binding of either fulvestrant or tamoxifen to the ER or trastzumab to HER2 receptor [Figure 1].

In the present review, we considered the relevance of exosomes as vehicles of prognostic and predictive markers as well as target molecules together with their potential therapeutic applications as drug cargo suppliers. We also attempted to clarify the association of their cargo with the initiation of drug resistance. In this respect, we performed an extensive literature search in PubMed, Web of Science, and Google Scholar, using the terms: “breast cancer”, “exosomes”, “exosomal”, “vesicles”, “therapy”, “response”, “resistance”, “predictive”, “prediction”, and “prognosis”. We found over 100 studies, of which most are cited with the exception of some studies that showed repetitive results.

## Therapy

For some years, targeted therapy has been increasingly applied in the management of breast cancer (BC). In this regard, neoadjuvant therapy has focused on combinations of systemic agents to optimize pathological complete response [Table 1].

**Table 1:** Drugs mainly used in breast cancer therapy and their function

Drug	Function	Ref.
Cisplatin, Carboplatin	Crosslinking of purine bases	[1,2]
Docetaxel, Paclitaxel	Microtubule stabilizer	[3,4]
Doxorubicin, Adriamycin	Intercalating of DNA	[5]
Epirubicin	Epimer of doxorubicin	[6]
Fulvestrant	Antiestrogen	[7]
Gemcitabine	Analogue of cytidine	[8]
Lapatinib	Dual tyrosine kinase inhibitor	[9]
Metformin	Glycerol-3-phosphate-dehydrogenase inhibitor	[10]
Olaparib	Poly-(ADP-ribose) polymerase inhibitor	[11]
Tamoxifen	Antiestrogen	[12]
Trastuzumab	HER2 inhibitor	[13]
Vinorelbine	Disruption of microtubules	[14]

HER2: human epidermal growth factor receptor 2

## Predictive value of exosomes and their cargo

**Figure 2:**
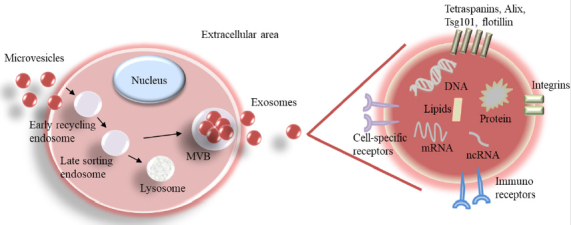
Biogenesis of exosomes and magnification of an exosome with its cargo. MVB: multivesicular body; ncRNA: noncoding RNA

A range of extracellular vesicles (EVs) has been observed to be released from both healthy and cancer cells. Their diameters range from 20-30 nm to 10 µm, the majority being smaller than 200 nm. They include ectosomes, exosomes, apoptotic bodies, large oncosomes, exophers, and enveloped viruses. The exosomal vesicles are identified by their size at 40-120 nm. Specific exosomal markers include Alix, Tsg101, tetraspanins (CD81, CD63, and CD9), and flotillin [Figure 2]^[15]^.

Exosomes are an important subgroup of vesicles. They act as a transport system to ferry material and informative molecules between cells over a range of distances. They appear to carry both genomic (100 bp-17 kb) and mitochondrial DNA, both cellular and mitochondrial-specific RNA (including mRNA, miRNA, rRNA, tRNA, lncRNA, piRNA, snRNA, and small nucleolar RNA), protein, ceramides, and cholesterol^[16-22]^. Specifically, they help cancer cells to propagate genetic information, possibly leading to the initiation of metastases. In addition, they are also important in moving chemotherapeutic drugs from cell to cell. This cell-to-cell communication facilitated by exosomes can aid the development of chemotherapy resistance. The other EV subgroups also seem to harbor these features, but exosomes are the best characterized subgroup.

The following paragraphs summarize the studies describing the exosome shuttle of nucleic acids and proteins and the biological activity of the released exosomal cargo molecules in the targeted recipient cells in the context of drug response and resistance in BC patients.

The biogenesis of exosomes starts with the endocytosis of microvesicles that fuse to an early endosome. Some early endosomes cycle back to the cell surface for elimination or further develop into late endosomes or lysosomes. During the formation of internal luminal vesicles (exosomes), late endosomes mature into MVBs (multivesicular bodies). The secreted exosomes contain DNA, RNA, protein, and lipids. On their surface, they present specific exosomal markers and cell-specific and immune-regulatory receptors.

### Exosomal DNA

The transferred exosomal DNA molecules affect the phenotype and function of the recipient cells, since the donor DNA is transcribed in these cells and, consequently, increases the levels of mRNA and protein^[23]^. So far, we could not detect any publication on the transfer of exosomal nuclear DNA fragments referring to drug resistance in BC patients, although there is one study on mitochondrial DNA (mtDNA).

Since mitochondria play a central and multifunctional role in cancer progression and metabolism^[24]^, Sansone *et al.*^[19]^ were interested in investigating whether or not the horizontal transfer of mtDNA mediates exit from dormancy and resistance to therapy. Functional analyses identified cancer-associated fibroblast (CAF)-derived EVs, including exosomes, to be loaded with the full mitochondrial genome in xenograft models of hormonal therapy-resistant metastatic BC. The treatment of hormone therapy-naive cells or hormone-treated metabolically dormant populations with these CAF-derived EVs promoted an escape from metabolic quiescence and hormonal therapy-resistance both *in vitro* and *in vivo*. These observations in multiple models of ER-positive BC indicate that CAFs could package high amounts of DNA into EVs, including even the full mitochondrial genome, which was then released and taken up by dormant cancer stem cells (CSCs). Subsequently, the foreign mtDNA was transcribed into mRNA, leading to either: (1) higher oxidative phosphorylation potential and exit from metabolic quiescence; or (2) dormancy of CSCs and the development of resistance to hormone therapy and therapy-resistant metastases. It should also be noted that exosomes can contain whole mitochondria (Wang *et al*.^[25]^ 2018), suggesting that when leukemia cells are challenged by chemotherapeutic drugs to induce oxidative stress, their dysfunctional mitochondria are transported in exosomes to neighboring bone marrow cells, thus leading to chemoresistance.

### Exosomal mRNA

Exosomes also contain different amounts of RNA involving more than a dozen different forms of RNA^[26]^, the majority of which are classified as noncoding RNAs (ncRNAs). They do not code for protein synthesis, but they are able to influence mRNAs. They can stabilize mRNA and may serve as shield against mRNA degradation. Equally, some forms can degrade mRNA. However, an important aspect is whether the mRNA molecules are intact or (partially) degraded in exosomes, since intact molecules can encode functional proteins and, thus, transfer functions of the cell of origin into the recipient cell, whereas partially degraded molecules may deliver new traits into the target cell^[27]^. To date, only a few publications have dealt with the transfer of exosomal mRNA referring to drug resistance in BC patients.

Cyclin-dependent kinase-4 and -6 (CDK4/6) play important roles in the cell cycle, which is usually dysregulated in cancer^[28]^. Therefore, targeted cancer therapies using CDK inhibitors are an attractive option to reestablish the cell cycle control. Palbociclib is an oral, first-in-class, highly selective CDK4/6 inhibitor that is applied in advanced and metastatic BC^[29]^. In their study, Del Re *et al.*^[30]^ analyzed plasma samples of 40 hormone receptor-positive/HER2-negative advanced BC patients before the administration of palbociclib plus endocrine therapy with fulvestrant. After three months, using digital droplet PCR, they quantified RNA isolated from exosomes and determined the expression levels of CDK4, CDK6, and CDK9 and thymidine kinase 1. They revealed that, in plasma-derived exosomes, high CDK4 mRNA levels were associated with a response to palbociclib plus fulvestrant, whereas increased levels of exosomal thymidine kinase 1 and CDK9 mRNA were associated with clinical resistance.

As documented by Boelens *et al*.^[31]^, communication of stromal cells with BC cells by the transfer of exosomes between both cell types can influence the treatment response. They demonstrated that exosomes transferred from fibroblasts to BC cells released their RNA and transposable elements into the target BC cells, thus resulting in the stimulation of the pattern recognition receptor retinoic acid-inducible gene I (RIG-I)^[32]^ with subsequent activation of the signal transducer and activator of transcription 1 dependent antiviral signaling pathway^[33]^. Crosstalk with the paracrine antiviral and juxtacrine NOTCH3 pathways^[34]^ led to regulation of the stroma-mediated propagation of therapy-resistant BC cells and reinitiated tumor growth. Analysis of primary human tissues and mouse basal-like BC models supported these cell culture experiments, which were abrogated by the combination therapy with gamma secretase inhibitors. These findings show that the microenvironment contributes to chemoresistance of BC cells^[31]^.

To reverse multidrug resistance (MDR) of adriamycin-resistant MCF-7 cells, Wang *et al.*^[35]^ investigated the potential role of exosomes in the treatment with psoralen, a naturally occurring compound with anti-cancer effects found in plants of the furocoumarin family^[36]^. In co-culture experiments, exosomes derived from adriamycin-resistant BC cells were able to promote active sequestration of drugs and induce a drug resistance phenotype in sensitive MCF-7 cells by transferring the drug-resistance-related gene MDR-1 mRNA. Psoralen reduced exosome production. KEGG pathway analysis showed that psoralen is involved in the peroxisome proliferator-activated receptor signaling pathway^[35]^ that regulates the synthesis of ceramide, an important regulatory molecule in exosomes secretion^[37]^.

### Exosomal microRNAs

As summarized in Table 2, chemoresistant BC cells secrete exosomes harboring different patterns of deregulated miRNAs. To date, a steadily growing body of publications has dealt with the propagation of chemoresistance by exosomal miRNAs. In the last decade, investigations on exosomes transferring miRNAs have been of central interest in the scientific world.

**Table 2:** Deregulation of exosomal miRNAs in breast cancer resistance

miRNAs	Target	Resistance	Ref.
miR-23a-3p	MAPK and Wnt signaling pathways	Adriamycin	[38]
miR-134	STAT5B	Cisplatin	[39]
miR-155	n.d.	Doxorubicin, paclitaxel	[40]
miR-155, miR-301	n.d.	Doxorubicin, paclitaxel	[41]
miR-23a, miR-222, miR-452	Sprouty2, PTEN, APC4	Docetaxel	[42]
miR-30a, miR-100, miR-222	n.d.	Adriamycin, docetaxel	[43]
miR-221/222	p27, ERα	Tamoxifen	[44]
miR-222	n.d.	Adriamycin	[45]
miR-370-3p, miR-373	n.d.	Cisplatin	[46]
miR-423-5p,	CCND2, CCND3	Cyclophosphamide, fluorouracil, epirubicin, docetaxel, paclitaxel	[47]
miR-574-3p	n.d	Docetaxel, epirubicin, vinorelbine	[48]
miR-770	Stathmin 1	Doxorubicin	[49]
miR-1246	CCNG2	Docetaxel, epirubicin gemcitabine	[50]
miR-9-5p, miR-195-5p, miR-203a-3p	ONECUT2	Docetaxel	[51]
miR-16, miR-23a, miR-24, miR-26a, and miR-27a	n.d.	Docetaxel	[52]

MAPK: mitogen-activated protein kinase; STAT5B: signal transducer and activator of transcription 5B; APC4: anaphase-promoting complex 4; ONECUT2: one cut homeobox 2; CCNG2: cyclin G2; CCND2: cyclin D2; CCND3: cyclin D3; ERα: estrogen receptor α; PTEN: phosphatase and tensin homolog deleted on chromosome ten

To evaluate the effect of EVs (exosomes) derived from triple-negative breast cancer (TNBC) cells on cytotoxicity to therapeutic agents in non-tumorigenic MCF10A, Ozawa *et al.*^[53]^ treated MCF10A cells with EVs derived from HCC1806 cells, a TNBC cell line. The EVs significantly increased cell proliferation and induced resistance to docetaxel and doxorubicin in MCF10A cells. Gene and miRNA expression profiling revealed 138 genes and 70 miRNAs significantly differentially expressed between EVs derived from the HCC1806 EV-treated MCF10A and the untreated MCF10A cells. The modulated exosome cargo derived from HCC1806 EV-treated MCF10A affected mostly the phosphatidyl inositol 3-kinase/AKT (PI3K/AKT)^[54]^, mitogen-activated protein kinase (MAPK)^[55]^, and hypoxia inducible factor 1 subunit alpha^[56]^ signaling pathways. Interestingly, EVs isolated from TNBC cells are capable of propagating drug resistance^[53]^.

Using microarray and PCR, Chen *et al.*^[38]^ exposed a signature of miRNAs differentially expressed between exosomes derived from adriamycin-resistant and parental sensitive BC cells. In adriamycin-resistant exosomes, the levels of 309 miRNAs were increased, whereas those of 66 miRNAs were decreased compared with sensitive BC cells. In particular, 52 miRNAs were overexpressed more than 16.0-fold in adriamycin-resistant exosomes. MiR-23a-3p was found to have the highest expression fold-change between resistant and sensitive exosomes. After predicting the target genes, 13 of 52 miRNAs inhibited target genes involved in MAPK and Wnt signaling pathways.

By profiling 384 miRNAs, O’Brien *et al.*^[39]^ showed that most exosomal miRNAs were down-regulated in aggressive BC cells. MiR-134 was the most substantially down-regulated miRNA. Using publicly-available tumor datasets, miR-134 was found to be significantly down-regulated in 77 breast tumor specimens derived from patients who underwent primary surgical treatment compared with 17 normal breast tissue specimens obtained from mammoplastic reduction. Functional analysis indicated that miR-134 inhibited signal transducer and activator of transcription 5B (STAT5B), which, in turn, controls Hsp90^[57]^. Transfection of miR-134 into Hs578Ts(i)8, a TNBC cell line with low levels of this miRNA decreased the levels of STAT5B, Hsp90, and Bcl-2, reduced cellular proliferation and increased cisplatin-induced apoptosis. Delivery via miR-134-enriched exosomes also decreased the levels of STAT5B and Hsp90, reduced cellular migration and invasion and increased sensitivity to anti-Hsp90 drugs^[39]^.

Circulating tumor cells and serum exosomal miRNAs were isolated from 53 BC patients before and at the middle of neoadjuvant therapy by Rodríguez-Martínez *et al.*^[58]^. Although the levels of exosomal miR-21, miR-222, and miR-155 were significantly associated with the presence of circulating tumor cells and the clinical parameters, their predictive role in response to chemotherapy was not significant^[58]^. Likewise, our laboratory did not find any association of cell-free (exosome-free) miR-21 with the pathological response, when we quantified miRNAs in serum of 127 HER2-positive BC patients before and after neoadjuvant therapy with trastuzumab and lapatinib using TaqMan microRNA assays^[59]^. These findings may imply that exosomal as well as cell-free miR-21 may be rather a diagnostic than a predictive marker in BC patients.

To date, miR-155 was described as an important epithelial-mesenchymal transition (EMT) and CSCs regulator^[60]^. In their study, Santos *et al.*^[40]^ demonstrated the relevance of miR-155 upregulation in chemoresistant cells associated with EMT. These researchers found increased levels of miR-155 in exosomes of CSCs and doxorubicin- and paclitaxel-resistant BC cells. Co-culturing exosomes derived from CSCs and chemoresistant cells with sensitive cells resulted in increased levels of miR-155 and induction of chemoresistance in the sensitive cells. Apart from these effects, EMT was observed in cells transfected with miR-155. These analyses show that exosomes loaded with miR-155 may transfer resistance and migration capacity to sensitive BC cells.

Using microarray cards, our laboratory quantified 45 selected exosomal miRNAs in plasma of 435 HER2-positive BC and TNBC patients undergoing treatment with paclitaxel and non-pegylated liposomal doxorubicin with or without addition of carboplatin. In uni- and multivariate models, miR-155 and miR-301 best predicted pathological response to chemotherapy. Our findings show a network of deregulated exosomal miRNAs with specific expression patterns in exosomes of HER2-positive and TNBC patients that are also associated with clinicopathological parameters and, in particular, with the pathological response within each BC subtype^[41]^.

Using microarray and PCR, Chen *et al.*^[42]^ found that exosomes from docetaxel-resistant BC cells contained a set of 20 most abundant miRNAs. Following transfer of exosomes derived from docetaxel-resistant cells to parental sensitive cells, the levels of some miRNAs were significantly increased and increased the overall resistance in the recipient cells. The exosomes were able to downregulate mRNAs, such as Sprouty2, involved in the MAPK pathway, phosphatase and tensin homolog deleted on chromosome ten, and anaphase-promoting complex 4 (APC4), an ubiquitin ligase, which are targeted by miR-23a^[61]^, miR-222^[62]^, and miR-452^[63]^, respectively.

In addition, Chen *et al.*^[43]^ showed that sensitive MCF-7 cells acquired increased survival potential from their adriamycin- and docetaxel-resistant BC variants by exosome transfer. Exosomes derived from adriamycin- and docetaxel-resistant MCF-7 cells significantly modulated the cell cycle distribution and drug-induced apoptosis in sensitive MCF-7 cells. When pre-treated with RNase, the exosomes were unable to affect these processes. Using microarray and PCR, the researchers found selective miRNA expression profiles in sensitive, adriamycin-derived, and docetaxel-derived exosomes. Target gene prediction and pathway analysis indicated the involvement of miR-30a, miR-100, and miR-222 in chemoresistance. The ability of miR-222 to mediate chemoresistance was confirmed by Wei *et al.*^[44]^. They compared the miRNA expression levels between tamoxifen-resistant and sensitive MCF-7 cells along with their exosomes. There were significant differences in the concentration and size distribution between exosomes derived from resistant and sensitive MCF-7 cells. Parental MCF-7 cells had higher amounts of exosomal RNA and proteins than resistant MCF-7 cells. Transmission of exosomes derived from resistant cells to sensitive MCF-7 cells led to the secretion of exosomal miR-222 as well as miR-221, and, in turn, reduced the expression of their target genes, namely the cyclin dependent kinase inhibitor p27^[64]^ and estrogen receptor α (ERα). This modulated expression pattern induced tamoxifen resistance in the recipient sensitive BC cells^[44]^. The acquired drug resistance of parental sensitive MCF-7 cells induced by miR-222 was also reported by Yu *et al.*^[45]^. It is likely that higher levels of miR-222 were detected in adriamycin-resistant MCF-7 cells co-cultured with exosomes derived from sensitive MCF-7 cells than in sensitive MCF-7 cells. Sensitive MCF-7 cells transfected with miR-222 mimics acquired adriamycin resistance, whereas sensitive MCF-7 cells transfected with miR-222 inhibitors blocked the resistance. These studies highlight the importance of miR-222 in the induction of chemoresistance.

To investigate the role of exosomes in chemoresistance to cisplatin, Wang *et al*.^[46]^ established cisplatin-resistant MDA-MB-231 cells. After incubation of sensitive MDA-MB-231 cells with exosomes derived from these cisplatin-resistant BC cells, the cells displayed a higher IC_50_ value for cisplatin, *P*-glycoprotein expression, migration and invasion capabilities, and a lower apoptosis rate. Using a microarray, 60 miRNAs in exosomes derived from cisplatin-resistant cells were significantly up-regulated when compared to exosomes from sensitive MDA-MB-231 cells. Notably, miR-370-3p, miR-423-5p, and miR-373 were the most differentially expressed miRNAs. Deregulation of exosomal miR-423-5p significantly affected resistance to cisplatin.

Using two different cell line types, Bovy *et al.*^[47]^ demonstrated that endothelial cells can transfer miRNAs to BC cells via exosomes. MiRNA profiling identified miR-503, which was downregulated in exosomes released from endothelial cells cultured under tumor conditions and which targeted the cyclins CCND2 and CCND3^[65]^. The modulation of miR-503 in BC cells altered their proliferative and invasive character. For the first time, Bovy *et al.*^[47]^ demonstrated the involvement of the endothelium in the modulation of tumor development via the secretion of circulating miR-503 in response to chemotherapy treatment.

Using microarray and RT-qPCR, Zhong *et al.*^[48]^ analyzed miRNA profiles in pre-neoadjuvant chemotherapy biopsies and paired surgically-resected specimens embedded in paraffin wax from 23 BC patients, as well as in three resistant sublines established by exposing the parental MDA-MB-231 cells to docetaxel, epirubicin, and vinorelbine, respectively. Exosomes harbored lower miRNA levels than cells; only the levels of 22 miRNAs were consistently up-regulated in exosomes. After pre-neoadjuvant chemotherapy, 12 of the 22 miRNAs were significantly up-regulated and might contribute to drug resistance of BC. The comparison of miRNA expression levels in the stable/progressive disease (SD/PD) BC subgroup with those in the partial response (PR) subgroup showed that only miR-574-3p in surgically-resected specimens was significantly up-regulated, suggesting a possible function of this miRNA in chemoresistance.

In the study by Li *et al.*^[49]^, the relevance of miR-770 in the regulation of chemoresistance and metastasis of TNBC patients was determined. The expression of miR-770 was higher in chemo-sensitive tissues than in chemo-resistant tissues, the high levels of this miRNA predicted a better prognosis in TNBC patients. Overexpression of miR-770 inhibited the resistance to doxorubicin in TNBC cell lines by regulating apoptosis and the tumor microenvironment. This process was mediated by exosomes and the targeting of the mRNA of Stathmin 1 (STMN1), a microtubule-destabilizing phosphoprotein particularly involved in the construction and function of the mitotic spindle^[66]^. Re-expression of STMN1 could partly reverse the chemo-sensitive effect of miR-770 in TNBC cells^[49]^.

To examine whether or not exosomal miR-1246 can be applied as a therapeutic agent, Li *et al.*^[50]^ measured the expression levels of miR-1246 in tumor tissues and serum of 56 BC patients. MiR-1246 was upregulated in BC patients compared with healthy women. The levels of miR-1246 were also significantly higher in metastatic BC MDA-MB-231 cells than in non-metastatic cells or non-malignant breast cells. Moreover, miR-1246 could inhibit the expression of its target gene, cyclin G2 (CCNG2), an inhibitor of cell-cycle progression^[67]^. Administration of exosomes derived from MDA-MB-231 cells could increase the viability, migration, and, in particular, resistance to chemotherapy of non-malignant HMLE cells^[50]^.

An adaptive mechanism by which communicating cancer cells survive in response to cytotoxic treatment was described by Shen *et al.*^[51]^. They demonstrated that chemotherapy induced BC cells to secrete exosomes containing miR-9-5p, miR-195-5p and miR-203a-3p. Inhibition of the expression of the transcription factor one cut homeobox 2 (ONECUT2) by these miRNAs the expression of stemness-associated genes, such as *NOTCH1*, *SOX9*, *NANOG*, *OCT4*, and *SOX2*, leading to the induction of the CSC character of the cells. Inversely, inhibition of these miRNAs, hence the re-expression of ONECUT2, quenched this CSC-stimulating effect. Likewise, in mice bearing xenograft mammary tumors, cytostatic treatment with docetaxel elevated the levels of miR-9-5p, miR-195-5p, and miR-203a-3p in circulating exosomes, decreased ONECUT2 expression, and increased stemness-associated gene expression. These effects were reduced in tumors deficient in exosome secretion and treated with neoadjuvant chemotherapy. Based on these results, the authors suggested that blocking the EV miRNA-ONECUT2 axis represents a potential strategy to maximize the deadly effect of chemotherapy and to reduce chemoresistance.

D-rhamnose β-hederin (DRβ-H), an active component extracted from the plant *Clematis ganpiniana*, is used in traditional Chinese medicine. It seems to be effective against BC by reducing the secretion of tumor-derived exosomes^[68]^. Chen *et al.*^[52]^ showed that, following absorption and internalization, exosomes from docetaxel-resistant BC MCF-7 cells spread resistance to recipient sensitive cells. DRβ-H could reduce the formation and secretion of these exosomes and was able to reverse the resistance to docetaxel. DRβ-H could also decrease the levels of several most abundant miRNAs (miR-16, miR-23a, miR-24, miR-26a, and miR-27a) that were transported by these exosomes and participate in pathways leading to treatment failure.

Finally, the selective packing of miRNAs into BC exosomes and the relevance of their delivery in the propagation of resistance to adriamycin was examined by Mao *et al.*^[69]^. The high total amounts of miRNAs in exosomes derived from adriamycin-resistant BC cells were significantly increased in recipient sensitive cells after the uptake of these exosomes. In particular, the adriamycin-derived exosomes contained diverse miRNAs associated with the Wnt signaling pathway^[70]^. Co-culture assays indicated that these exosomes were able to induce the overall resistance to adriamycin in sensitive BC cells^[69]^.

### Exosomal lncRNAs

Despite the identification of myriad lncRNAs, thus far few studies have dealt with the relevance of exosomal lncRNAs in BC chemoresistance [Table 3].

**Table 3:** Deregulation of exosomal lncRNAs in breast cancer resistance

lncRNAs	Target	Resistance	Ref.
HOTAIR	n.d.	Tamoxifen	[71]
AGAP2-AS1	n.d.	Trastuzumab	[72]
SNHG14	BAX	Trastuzumab	[73]
UCA1	n.d.	Tamoxifen	[74]

SNHG14: small nucleolar RNA host gene 14; UCA1: urothelial carcinoma-associated 1; BAX: B-cell lymphoma 2 associated X protein; n.d.: not determined

In their study, Tang *et al.*^[71]^ investigated the diagnostic and prognostic value of HOTAIR in exosomes derived from the serum of BC patients undergoing neoadjuvant chemotherapy and tamoxifen therapy. Higher serum levels of exosomal HOTAIR were detected in BC patients than in healthy women. Three months after surgery, the levels of exosomal HOTAIR decreased compared with those before surgery. High expression of exosomal HOTAIR resulted in a worse disease-free survival and overall survival. In the high-expression chemotherapy subgroup, six BC patients achieved a partial response (PR) and eight had a stable disease (SD), while nine patients achieved a PR and two had a SD in the low-expression group. In the low-expression subgroup treated with tamoxifen, one patient had a recurrence and the other ten patients had no recurrence, while six had a recurrence and seven had no recurrence in the high expression group. These findings point to a potential association of high serum levels of exosomal HOTAIR with poor neoadjuvant chemotherapy and response to tamoxifen therapy.

To reveal the function of lncRNA AGAP2-AS1 and its underlying regulatory mechanism in resistance to trastuzumab, Zheng *et al.*^[72]^ quantified AGAP2-AS1 in parental and trastuzumab-resistant HER2-positive SKBR-3 and BT474 cells. They found that AGAP2-AS1 was upregulated in the resistant cells when compared with the parental sensitive cells. A cell viability assay showed that silencing of AGAP2-AS1 increased the cytotoxicity induced by trastuzumab treatment. Co-culturing exosomes containing AGAP2-AS1 with sensitive cells reduced the cell death induced by trastuzumab, whereas knockout of exosomal AGAP2-AS1 reversed this effect.

Using a lncRNA microarray assay followed by RT-qPCR, Dong *et al.*^[73]^ identified potential exosomal lncRNAs during the development of chemoresistance in BC. In particular, the small nucleolar RNA host gene 14 (*SNHG14*) was upregulated in trastuzumab-resistant cells when compared with parental BC cells. Functional experiments demonstrated that silencing of this lncRNA promoted trastuzumab-induced cytotoxicity. SNHG14, which was incorporated into exosomes, propagated trastuzumab resistance to sensitive cells. A signal transduction reporter array indicated that this lncRNA exerted its effect by targeting the Bcl-2 apoptosis regulator B-cell lymphoma 2 associated X protein (BAX) signaling pathway^[75]^. Furthermore, the serum levels of exosomal SNHG14 were significantly higher in 34 advanced HER2-positive BC patients with resistance to trastuzumab than in 38 BC patients who responded to the therapy^[73]^.

The differing load of urothelial carcinoma-associated 1 (UCA1) in exosomes between tamoxifen-sensitive and -resistant BC cells was described by Xu *et al.*^[74]^. The levels of UCA1 were significantly higher in tamoxifen-resistant LCC2 cells than in tamoxifen-sensitive MCF-7 cells. High amounts of UCA1 were also loaded into exosomes secreted by tamoxifen-resistant LCC2, a subline of MCF-7 cells. The exosome mediated transfer of UCA1 from LCC2 cells could increase tamoxifen resistance in MCF-7 cells. MCF-7 cells co-cultured with LCC2-derived exosomes also had an increased cell viability, a decreased expression of cleaved caspase-3, and a lower ratio of apoptosis after tamoxifen treatment. LCC2-derived exosomes with impaired UCA1 loading had a minor ability to promote tamoxifen resistance in MCF-7 cells, implying the involvement of UCA1 in resistance to tamoxifen.

### RNA epitranscriptomes

Since 1975, when Adams and Cory^[76]^ demonstrated the presence of N6-methyl adenosine and new 5’ termini bearing 7-methyl-guanosine in a 5’,5’triphosphate linkage with ribose methylated nucleosides, epitranscriptome changes to mRNA in mouse myeloma cells have been known. Various additional common 5’-terminal sequences of the forms m7G5’ppp5’NmpNp and m7G5’ppp5’NmpNmpNp were also observed. This work did not stimulate further studies until much later, in part, due to the lack of sufficiently sensitive techniques at that time.

The current situation accepts that mRNA, tRNA, rRNA, and ncRNAs can be epigenetically modified, such RNA modifications being considered to be tightly regulated and having a central role in the control of both mRNAs and ncRNAs^[77-79]^. The controls include the efficiency of mRNA stability and translation^[80,81]^. M6A mRNA methylation is an important factor in the promotion of reprogramming to pluripotency and from naïve pleuripotency to differentiation^[82,83]^. M6A appears to be the most common and abundant transcriptional modification of the mRNAs. M6A modifications are applied by the m6A methyltransferases including METTL3/14, WTAP, RBM15/15B and KIAA1429. These are referred to as “writers”, M6A removal is made by the demethylases FTO and ALKBH5 that are known as “erasers”. Finally, recognition is by the m6A binding proteins YTHDF1/2/3, IGF2BP1, and HNRNPA2B1 that are called “readers”^[84]^.

Such modifications to mRNAs and ncRNAs have also been recorded for cancers^[79]^. M6A RNA alterations seem to regulate RNA transcript, splicing, processing, translation, and decay as well as participating in both tumorigenesis and metastasis of a variety of malignancies^[84-87]^. In the case of BC, when associated with the expression of mammalian hepatitis B X-interacting protein (HBXIP), METTL3 shows an aggressiveness in BC promotion through the inhibition of tumor suppressor let-7g^[88]^.

These, among others, results relating to the involvement of m6A modified mRNAs imply that this field should be further studied in conjunction with the various aspects of BC exosomes and treatment resistance.

### Exosomal proteins

Besides nucleic acids, exosomes may carry proteins, of which about 10% of the total exosomal protein content may be of mitochondrial origin^[89]^ including some exosomes carrying the mitochondrial electron transport chain complexes^[90]^. Their function is not clear. Thus, functional proteins participating in diverse signaling pathways leading to drug resistance are selectively packaged into exosomes. These oncogenic proteins are also protected by exosomes from agents that target these proteins.

Exosomes constitutively secreted by HER2-overexpressing BC cell lines were characterized and their potential role in interfering with the therapeutic activity of trastuzumab and lapatinib was analyzed *in vitro* and *in vivo* by Ciravolo *et al.*^[91]^. The researchers showed that exosomes released by the HER2-overexpressing tumor cell lines SKBR3 and BT474 also expressed HER2 molecules. These exosomes secreted either in HER2-positive tumor cell-conditioned supernatants or in serum of BC patients bound to trastuzumab and inhibited trastuzumab activity on SKBR3 cell proliferation, suggesting the role of HER2-positive exosomes in modulating sensitivity to trastuzumab. Surprisingly, the lapatinib activity on SKBR3 cell proliferation was unaffected by the presence of autologous exosomes. Similar observations were made by Hung *et al.*^[92]^ who used a TNBC model. They showed that EV-encapsulated epidermal growth factor receptor (EGFR) is protected from inhibitors that target EGFR and can activate a signaling pathway in recipient BC cells, to promote proliferation and migration ability *in vitro*. This mechanism provokes the EVs, such as exosomes, to be a target shelter, which, when released, can cause drug resistance and support tumor progression in the recipient BC.

In their study, Ning *et al.*^[93]^ demonstrated that adriamycin-resistant MCF-7 cells secreted exosomes containing ubiquitin carboxyl terminal hydrolase-L1 (UCH-L1)^[94]^ and *P*-glycoprotein into the extracellular microenvironment. The adriamycin-sensitive BC cells took up these exosomes in a time-dependent manner, and adopted a chemoresistant phenotype. Remarkably, the UCH-L1 levels in circulating exosomes of 47 chemotherapy responders were significantly lower than those from 46 non-responders. These findings show a negative correlation of the levels of exosomal UCH-L1 with chemotherapy outcome^[93]^. Lv *et al.*^[95]^ also investigated the cell-to-cell communication by exosomes containing *P*-glycoprotein. However, they established and used a docetaxel-resistant variant of MCF-7 cells. Sensitive MCF-7 cells, co-cultured with exosomes that were extracted from docetaxel-resistant MCF-7 cells, increased the levels of *P*-glycoprotein along with their up-take of exosomes to acquire a *P*-glycoprotein expression pattern similar to the resistant cells that resulted in drug resistance.

Transient receptor potential channel (TRPC5) plays an important role in the induction of *P*-glycoprotein in drug-resistant BC cells^[96]^. To examine whether or not TRPC5 can serve as a noninvasive biomarker for the imaging examination of chemoresistance, Wang *et al.*^[97]^ analyzed plasma from 131 BC patients and the corresponding tumor tissue from 54 patients before anthracycline/taxane-based chemotherapy. They found that circulating exosomes contained TRPC5. The levels of exosome-carrying TRPC5 were significantly associated with the expression of TRPC5 in BC tissues and tumor response to chemotherapy. Furthermore, the increased levels of exosomal TRPC5 after chemotherapy preceded partial response, and predicted acquired chemoresistance.

Neuromedin U (NmU), a neuropeptide, has multiple functions in different organs and tissue types^[98]^. It plays a critical role in HER2-positive BC, associating with aggressiveness, resistance to HER2-targeted therapies, and poorer outcome^[99]^. To elucidate the mechanism through which NmU exerts these effects, Martinez *et al.*^[100]^ used HER2-positive BC cells that stably over-expressed NmU. These cells and their shed exosomes harbored elevated yields of the immunosuppressive cytokine tumor growth factor β1 (TGFβ1) and the lymphocyte activation inhibitor PD-L1. More importantly, the cells displayed an increasing resistance to antibody-dependent cell cytotoxicity mediated by trastuzumab, suggesting a role of NmU in enhancing immune evasion. These traits were also detected in HER2-targeted drug-resistant cells that expressed higher levels of NmU than their drug-sensitive counterparts. Strikingly, exosomes derived from resistant cells were able to increase the levels of TGFβ1 in drug-sensitive cells. Moreover, in the serum of HER2-positive patients who did not respond to HER2-targeted drug treatment with trastuzumab with or without lapatinib, TGFβ1 levels were significantly higher in exosomes than in patients who had a complete response (CR) or partial response (PR), indicating a possible new mechanism-of-action for NmU in HER2-positive BC that increases resistance to the anti-tumor immune response.

Surprisingly, experimental studies in mouse mammary tumor models imply that chemotherapy has pro-metastatic effects^[101]^. Keklikoglou *et al.*^[102]^ disclosed that two classes of cytotoxic drugs, taxanes and anthracyclines used in neoadjuvant BC therapy elicited tumor-derived EVs, including exosomes, with increased pro-metastatic capacity. Chemotherapy-elicited EVs were enriched in annexin A6 (ANXA6), a Ca^2+^-dependent protein^[103]^ that promoted NF-κB-dependent endothelial cell activation and induced cytokine Ccl2 and Ly6C^+^CCR2^+^ monocyte expansion^[104]^ in the pulmonary pre-metastatic niche to facilitate the formation of lung metastasis. Genetic inactivation of ANXA6 reduced the pro-metastatic effects of chemotherapy-elicited EVs^[101]^.

Glutathione S-transferase P1 (GSTP1), which belongs to the family of phase II metabolic enzymes, detoxifies anti-cancer drugs by conjugating them with glutathione^[105]^. To clarify the mechanisms that regulate the GSTP1-dependent drug resistance, Yang *et al.*^[106]^ investigated the role of GSTP1-containing exosomes. Analyses of 42 paired BC tissues collected before and after anthracycline/taxane-based neoadjuvant chemotherapy exhibited higher GSTP1 expression in the PD/SD subgroup than in the PR/CR subgroup in the samples collected both before and after chemotherapy. Analyses of exosomes from the corresponding serum of 30 BC patients treated with the neoadjuvant chemotherapy also showed higher GSTP1 levels in the PD/SD subgroup than in the PR/CR subgroup. Interestingly, increased levels of GSTP1 and the exosomal marker tumor susceptibility gene 101 protein (TSG101) were observed in the cytoplasm after chemotherapy. These findings point to the ability of GSTP1-containing exosomes to transfer drug resistance.

As documented by Semina *et al.*^[107]^, co-culturing estrogen-dependent MCF-7 cells with MCF-7 sublines resistant to tamoxifen and/or biguanide metformin resulted in the partial resistance of the MCF-7 cells to the antiestrogen drugs within 14 days. In addition, there was a decrease in ERα activity and a parallel activation of the Akt signaling pathway^[108]^ as well as the transcription factors activator protein-1 (AP-1), nuclear factor-κB (NF-κB)^[109]^, and SNAIL1, a regulator of EMT^[110]^.

When Kreger *et al.*^[111]^ treated the highly aggressive BC cell line MDAMB231 with paclitaxel, these cells produced exosomes highly enriched with the cell survival protein survivin, a member of the family of apoptosis inhibitory proteins^[112]^. These exosomes promoted the survival of serum-starved and paclitaxel-treated fibroblasts and SKBR3 BC cells, an effect that could be blocked when survivin was silenced by siRNA in these vesicles. Thus, the enrichment of a specific cargo in exosomes may serve as a marker of paclitaxel resistance^[111]^.

## Exosomes as drug delivery systems for therapeutics

Exosomes are particularly qualified for the use as drug delivery systems and have advantages over other systems. This is owed to their characteristics since they are cell-derived, carry the membrane of the cell of origin with a natural composition, and are small and immune suppressive. There are different technologies to load exosomes with exogenous cargoes^[113]^, e.g., introduction of drugs into extracted, purified exosomes by electroporation, loading of donor cells with drugs, or transfection of donor cells with DNA encoding for a drug with subsequent extraction of the exosomes released from the cells into the medium^[114]^.

### Derived from BC cells

An exosome platform that can target regions of tumor hypoxia and be monitored *in vivo* using magnetic particle imaging was developed by Jung *et al.*^[115]^. Four types of exosomes were generated from MDA-MB-231 cells under hypoxic or normoxic conditions, as well as with or without exposure to X-ray radiation. They were modified to carry superparamagnetic iron oxide nanoparticles and olaparib. Fluorescence-activated cell sorting (FACS) and fluorescence microscopy showed that hypoxic cells preferentially took up exosomes released by hypoxic cells when compared to the other exosomes. *In vivo*, the therapeutic efficacy of olaparib-loaded exosomes was confirmed by increased apoptosis and slower tumor growth.

Trastuzumab emtansine (T-DM1) is an antibody-drug conjugate that carries a cytotoxic drug (DM1) to HER2-positive BC. T-DM1 binds to exosomes derived from HER2-positive BC cells, but not to exosomes derived from HER2-negative MCF-7 cells^[116]^. Barok *et al.*^[117]^ showed that HER2-positive SKBR-3 BC cells accumulated T-DM1 following administration of T-DM1-containing exosomes, resulting in growth inhibition and activation of caspases 3 and/or 7 in the apoptosis pathway.

As documented by Wang *et al.*^[118]^, MCF7 cells in exponential growth phase released about 220 exosomes per cell into the culture medium. In a dose- and time-dependent manner, paclitaxel and doxorubicin stimulated this exosome release. Exosomes isolated from donor cells treated with paclitaxel caused cytotoxicity and inhibited migration of recipient cells.

### Derived from dendric cells

A doxorubicin delivery platform using engineered exosomes was developed by Tian *et al.*^[119]^. This laboratory was able to deliver doxorubicin to tumor tissue in BALB/c nude mice. To reduce immunogenicity and toxicity, mouse immature dendritic cells were used for exosome production. Tumor targeting was facilitated by engineering the immature dendritic cells to express the exosomal membrane protein Lamp2b fused to a αv integrin-specific integrin-binding arginine-glycine-aspartic acid (iRGD) peptide. Applying electroporation, the purified exosomes were loaded with doxorubicin. The intravenously injected exosomes delivered doxorubicin specifically to tumor tissues in BALB/c nude mice, thus leading to the inhibition of tumor growth without overt toxicity.

### Derived from mesenchymal stem cells

Mesenchymal stem cells (MSCs) are multipotent cells that can be isolated from bone marrow, adipose tissue, umbilical cord, and placenta. Because of their immune suppressive and anti-inflammatory properties and high capacity of expansion *in vitro*, they are largely used in therapy^[120]^. To date, exosomes have previously been described as the main therapeutic factor in MSC secretion to promote immunomodulatory and regenerative abilities of MSC in tissue repair^[121]^.

Following administration of MSC populations with sub-lethal concentrations of taxol for 24 h, Melzer *et al.*^[122]^ isolated the taxol-loaded MSC-released exosomes. Co-culturing of these exosomes with highly metastatic MDA-hyb1 BC cells led to 80%-90% cytotoxicity of the BC cells. In addition, the researchers introduced the MDA-hyb1 cells into NODscid mice. A subsequent systemic intravenous application of MSC-derived taxol-loaded exosomes resulted in a more than 60% reduction of subcutaneous primary tumors and a 50% reduction of distant organ metastases in lung, liver, spleen, and kidney of these mice. These effects were similar to the effects with free taxol, although the concentration of taxol in exosomes was about 1000-fold reduced. Thus, the application of taxol-loaded MSC-derived exosomes might improve patients’ tolerance to taxol.

Likewise, Kalimuthu *et al.*^[123]^ extracted paclitaxel-loaded exosomes from bone marrow MSC cultured with paclitaxel. The anticancer effects of these exosomes were assessed with MDA-MB-231 cells both *in vitro* and *in vivo*. Increasing concentrations of the exosomes decreased the viability of MDA-MB-231 cells *in vitro*, while *in vivo* the tumor growth was significantly inhibited.

Applying electroporation, Gomari *et al.*^[124]^ encapsulated doxorubicin into exosomes extracted from bone marrow MSC. Flow cytometry revealed that 46% of targeted exosomes bound to HER2-positive cells, whereas 14% of exosomes bound to HER2-negative cells, suggesting a preferential cellular uptake of these exosomes by HER2-positive cells relative to HER2-negative cells^[125]^. The determination of cell viability by the MTT assay showed that the cytotoxicity of doxorubicin-loaded exosomes was higher than that of free doxorubicin at 72 h, suggesting a specific delivery of doxorubicin by targeted exosomes. A significant reduction of tumor growth rate was observed in a murine BC model^[124]^.

### Macrophage-derived exosomes

The suitability of M1 macrophage-derived exosomes as drug vesicles and their effect on the transcription factor NF-κB was examined by Wang *et al.*^[126]^. They showed that M1 macrophage-derived exosomes established a pro-inflammatory environment, which enhanced the anti-tumor activity via the caspase-3 mediated apoptosis pathway. The treatment of tumor bearing mice with M1 macrophage-derived exosomes caused anti-tumor effects. Moreover, paclitaxel carrying M1 macrophage-derived exosomes displayed higher anti-tumor effects than untreated M1 macrophage-derived exosomes or free paclitaxel.

In summary, the above studies document the higher anti-tumor efficacy of exosomes loaded with chemotherapeutic drugs rather than the use of free drugs, although the exosomes contain much lower concentrations of drugs. This better anti-tumor effect is due to a higher affinity of exosomes to their target tumors since they can be engineered to carry surface markers specific for guiding them to their destination. Their clinical use in the future is promising and suggests a better tolerance by patients treated with an exosome-based therapy rather than with directly used compounds.

## Exosomes as therapeutics

Since exosome levels are usually elevated in different cancer types^[41,127]^, exclusion of exosomes from the blood circulation or their reduction to normal levels has been proposed as a new therapeutic strategy to prevent poor outcomes of cancer patients. In this regard, the biotechnology company Aethlon Medical Inc. (San Diego, CA, USA) developed an adjunct therapeutic approach using an affinity plasmapheresis platform that decreases systemic secretion of HER2-positive exosomes by tumors^[128]^. On the other hand, tumor-derived exosomes carry antigens on their surface and can be used as a source for specifically stimulating the immune response against cancer^[129]^. As detailed reviewed by Rashed *et al.*^[130]^, exosomes can be used either as therapeutic target molecules or as agents.

Although therapies with anti-HER2 monoclonal antibodies have improved the clinical outcome, HER2-positive BC patients develop resistance to the antibody therapy. Therefore, HER2-specific vaccines, e.g., antigens carrying exosomes that may increase the immune stimulatory responses in these patients, have been established. In this regard, Li *et al.*^[131]^ developed a new HER2-specific exosome vaccine using polyclonal CD4-positive T cells prepared with exosomes derived from HER2-specific dendritic cells and demonstrated its therapeutic efficacy to reduce HER2-positive tumors in double-transgenic HER2/HLA-A2 mice with a HER2-specific self-immune tolerance. The same laboratory^[132]^ advanced this vaccine by constructing an adenoviral vector encoding a fusion protein composed of HER2 fragments, and developed a heterologous human/rat HER2-specific exosome-targeted T-cell vaccine. The researchers used polyclonal CD4-positive T-cells that took up exosomes released by dendritic cells that were transfected with the created adenoviral vector. This vaccine stimulated better CD4-positive T-cell responses, leading to a higher induction of HER2-specific antibodies (~70 µg/mL) than the former vaccine (~40 µg/mL), suggesting that this vaccine may be a new therapeutic alternative for patients with trastuzumab-resistant HER2-positive BC. Currently, exosomes as therapeutic agents have also been generated to treat TNBC, as reviewed by Li *et al.*^[133]^.

In their study, Walker *et al.*^[134]^ investigated the association of macrophages (MΦs) within the bone marrow stroma with dormancy. MΦs that exhibited an M2 phenotype and constituted about 10% of cultured bone marrow stroma activated BC dormancy, creating gap junctional intercellular communication with CSCs and leading to reduced proliferation and carboplatin resistance. In contrast, MΦs exhibiting the M1 phenotype reversed BC dormancy. Direct activation of M2a MΦs via the toll-like receptor 4 or indirectly through activation of MSC switched M2a MΦs to M1 phenotype. M1 MΦ-derived exosomes activated NF-κB to reverse quiescence of the circulating BC cells. In an *in vivo* model of BC dormancy, injected M2a MΦs sensitized the BC cells to carboplatin and increased their overall survival.

In summary, these investigations demonstrate that, based on the complex biology of exosomes and their role as communicating mediators among cells, exosomes are promising therapeutic structures/organelles to improve anticancer therapy.

## Isolation and characterization methods of exosomes

There are various methods to isolate exosomes^[135]^. The most commonly used technique for exosome isolation is ultracentrifugation. However, this method is time-consuming and requires expensive equipment. Remaining impurities and decreased yield of the isolated exosomes can be caused by the similarity in sedimentation properties of the different EV subgroups, by applying the same protocols for different types of rotors, and without considering the sample viscosity. In addition, ultracentrifugation can lead to exosomal damage^[136]^. The second method of choice for exosomes separation is based on the precipitation with polyethylene glycol. Although this technique is fast, easy, and non-laborious, it struggles with contaminations of plasma proteins and precipitation chemicals. One of the simplest methods of exosomes isolation is ultrafiltration. Ultrafiltration is a membrane-based technique of separating particles according to their size or molecular weight. However, this method is not efficient for diluted input material, and the extracted exosomes are highly diluted and display protein complexes in the exosomal fractions. An exemplary application of an affinity capture is the use of magnetic beads conjugated to monoclonal antibodies specific to proteins exposed on an exosomal membrane. Here, the efficiency of antibodies should be considered. The quantity and integrity of exosomes isolated can be determined by Western blot, Nanoparticle Tracking Analysis, and microscopy. For example, Western blot can be carried out by using antibodies specific for exosomal surface proteins, such as annexins, tetraspanins (CD9, CD63, CD81, and CD82), and heat-shock proteins (Hsp60, Hsp70, and Hsp90)^[135]^. However, all these methods require improvements and standardization for the daily clinical practice.

## Conclusion

Drug resistance is a major obstacle for a successful BC treatment. Therefore, a wealth of publications deals with drug response/resistance in cancer patients, including BC patients, and searches for means to overcome it. Concerning this matter, the liquid biopsy^[137,138]^ is a promising research field that relies on the analyses of circulating nucleic acids and exosomes. The advantage of analyzing exosomes is the fact that exosomes contain DNA, RNA, and protein profiles that they efficiently propagate among distant cells. Identification of this exosome cargo along with the verification of its transfer from cell to cell has the potential for a better understanding of the development of drug resistance, as well as for assessing predictive biomarkers for monitoring the efficacy of the treatment regimen. Although the current studies have recommended the clinical application of exosomes as agents or target molecules, the consequences of such an exosomes-based therapy remain unknown. Of therapeutic interest may be the inhibition of exosome secretion from cancer cells. Small hairpin interfering RNA (ShRNA) silencing of the GTPases Rab27a/b and using an inhibitor of sphingomyelinase (GW4869) or annexin/diannexin represent potential strategies to prevent the promoting effects of exosomes on tumor progression, angiogenesis, and metastasis^[139,140]^. Whether it is advisable to reduce exosomes to normal levels or even remove them from the bloodstream is not predictable. We know too little about the complex biology and function of exosomes. Exosomes act either as guardians to support systemic prosperity or as a mediator to propagate disease and drug resistance. These controversial characteristics make it challenging to use exosomes as target molecules. On the other hand, exosomes may be efficient drug vesicles and their cargo provides information on the stage of disease. Thus, further research is now warranted to increase our knowledge of the mechanism of cellular communication mediated by exosomes in order to develop future exosome-based therapeutics and overcome drug resistance. Identifying genomic profiling in exosomes during development of drug resistance has the potential to assess various biomarkers for inhibiting this negative development and change the therapy arm. It is also essential to develop technologies to deliver exosomes with a therapeutic content to target tissues. A potential strategy could be the use of genetically modified MSC for the delivery of such exosomes.

## Declarations

### Copyright

© The Author(s) 2020.
